# The Impact of Monitoring Depth of Anesthesia and Nociception on Postoperative Cognitive Function in Adult Multiple Trauma Patients

**DOI:** 10.3390/medicina57050408

**Published:** 2021-04-23

**Authors:** Ana-Maria Cotae, Mirela Ţigliş, Cristian Cobilinschi, Alexandru Emil Băetu, Diana Maria Iacob, Ioana Marina Grinţescu

**Affiliations:** 1 Anaesthesia and Intensive Care Clinic, Clinical Emergency Hospital of Bucharest, 014461 Bucharest, Romania; Mirelatiglis@gmail.com (M.Ţ.); cob_rodion@yahoo.com (C.C.); alexandru.baetu@gmail.com (A.E.B.); diana.iacob10@gmail.com (D.M.I.); ioana.grintescu@rospen.ro (I.M.G.); 2 Department of Anesthesia and Intensive Care, Faculty of Medicine, University of Medicine and Pharmacy Carol Davila, 050474 Bucharest, Romania

**Keywords:** entropy, POCD, general emergency surgery, anesthesia depth

## Abstract

*Background and Objectives*: Patients with traumatic injuries have often been excluded from studies that have attempted to pinpoint modifiable factors to predict the transient disturbance of the cognitive function in the postoperative settings. Anesthetists must be aware of the high risk of developing postoperative delirium and cognitive dysfunction (POCD) in patients undergoing emergency surgery. Monitoring the depth of anesthesia in order to tailor anesthetic delivery may reduce this risk. The primary aim of this study was to improve the prevention strategies for the immediate POCD by assessing anesthetic depth and nociception during emergency surgery. *Material and Methods*: Of 107 trauma ASA physical status II–IV patients aged over 18 years undergoing emergency noncardiac surgery, 95 patients were included in a prospective randomized study. Exclusion criteria were neurotrauma, chronic use of psychoactive substances or alcohol, impaired preoperative cognitive function, pre-existing psychopathological symptoms, or expected surgery time less than 2 h. Entropy and Surgical Pleth Index (SPI) values were constantly recorded for one group during anesthesia. POCD was assessed 24 h, 48 h, and 72 h after surgery using the Neelon and Champagne (NEECHAM) Confusion Scale. *Results*: Although in the intervention group, fewer patients experienced POCD episodes in comparison to the control group, the results were not statistically significant (*p* < 0.08). The study showed a statistically significant inverse correlation between fentanyl and the NEECHAM Confusion Scale at 24 h (r = −0.32, *p* = 0.0005) and 48 h (r = −0.46, *p* = 0.0002), sevoflurane and the NEECHAM Confusion Scale at 24 h (r = −0.38, *p* = 0.0014) and 48 h (r = −0.52, *p* = 0.0002), and noradrenaline and POCD events in the first 48 h (r = −0.46, *p* = 0.0013 for the first 24 h, respectively, and r = −0.46, *p* = 0.0002 for the next 24 h). *Conclusions*: Entropy and SPI monitoring during anesthesia may play an important role in diminishing the risk of developing immediate POCD after emergency surgery.

## 1. Introduction

First described in the mid-20^th^ century, cognitive dysfunction following anesthesia and surgery is a complication that can have a significant impact on patients, leading to unfavorable outcomes [1]. Postoperative cognitive dysfunction (POCD) is described as a decline of the intellectual functions and processes (both basic and higher executive skills) that develops after surgery [2]. Although recognized as a transient decline of cognitive function, POCD can persist for weeks, months, or more. POCD also interferes with patients’ psychological status, long-term outcome, mortality, and hospital discharge [3,4,5].

Postoperative cognitive decline occurs more frequently in the elderly population, with a higher incidence in patients older than 60 years irrespective of the type of anesthesia and surgery. Despite the fact that studies assessing cognitive impairment have been primarily centered on the study of older patients, there is a general agreement that POCD is more likely to occur after major surgery [5,6]. Although this type of cognitive dysfunction is considered multifactorial, it remains difficult to determine whether its occurrence is a result of patient-, surgical-, or anesthesia-related factors [7]. Several risk factors have been suggested to be involved in the pathophysiology of cognitive dysfunction, such as inflammatory cytokines, pain, preoperative impairment in neurocognitive function, metabolic disturbances, duration/type of surgery, hypoxemia, old age, and the use of certain anesthetics (sedation medication or different volatile anesthetic agents) [5,6,7,8].

Fortunately, several screening tests are available to establish cognitive disorders. Among these, one stands out for being easily performed without supplemental training. The Neelon and Champagne (NEECHAM) Confusion Scale was developed not only to identify postoperative delirium but also to classify patients as “early to mild confused ”, “at risk”, or “normal ” [9,10]. A score between 0 and 24 points is conclusive for the presence of at least one cognitive impairment. The scale has acceptable sensitivity, specificity, and predictive values when compared to the CAM-ICU [11].

This recent development encourages us to use electroencephalography (EEG) monitoring to assess the depth of anesthesia. Using neuromonitoring during anesthetic delivery can reduce the risk for postoperative cognitive side effects [12]. Among the anesthesia monitors currently approved to assess the depth of anesthesia, the entropy monitor proves to be one of the most reliable. The entropy device is capable of acquiring not only EEG signals but also frontal electromyography data, transforming them into two values: State and response entropy. The entropy device then displays state and response entropy as numerical values, denoting the depth of anesthesia. State entropy and response entropy are given indices between 0–91 and 0–100, respectively, ranging from complete suppression of cortical neuronal activity to an awake-state EEG [13].

As we have already mentioned, another important element in developing POCD is pain. In order to monitor intraoperative nociceptive stimulation and antinociceptive drug effects, different tools have been proposed over the years. Among them, the Surgical Pleth Index (SPI) has received recognition after several studies reported a better outcome in comparison to conventional analgesia. The Surgical Pleth Index module is designed to acquire and process the plethysmograph pulse wave and heartbeat frequency. The parameter has a range of value between 0 and 100. Although there is little to no validation of a specific cut-off value, previous studies have recommended a target value of SPI ≤ 50 [14,15].

The aim of this study was to reduce the incidence of POCD in the first 72 h by assessing anesthetic depth using entropy and nociception through the Surgical Pleth index (SPI) during emergency surgery.

## 2. Materials and Methods

### 2.1. Study Design

This prospective randomized study was carried out in the Anaesthesia and Intensive Care Clinic, Clinical Emergency Hospital of Bucharest, between August 2018 and January 2019. All the procedures performed during this study were in accordance with the Declaration of Helsinki.

The study was approved by the Research Ethical Committee of our hospital (registration number 2100/2021), and all the patients provided written informed consent. Patients were considered eligible for the study if they were over 18 years old, undergoing emergency noncardiac surgery expected to last at least 2 h, and American Society of Anesthesiologists (ASA) physical status II, III, or IV. The surgical procedures included abdominal (splenectomy, splenorrhapy, hepatorrhapy, hemicolectomy, phrenoraphy) and orthopedic (femoral osteosynthesis, tibial osteosynthesis, humeral osteosynthesis) surgery. Exclusion criteria were neurotrauma, chronic use of psychoactive substances or alcohol, impaired preoperative cognitive function pre-existing psychopathological symptoms, neurological deficits, or expected surgery time less than 2 h. From the collection data process, we excluded patients intubated prior to the surgical procedure and those who remained intubated at the end of the surgical procedure. The patients were consecutively assigned into 2 study groups. In the first group, anesthesia was provided under standard monitoring (SMG): 5-lead electrocardiogram, noninvasive arterial pressure, pulse oximetry, temperature and end-tidal carbon dioxide concentration. In the second group, apart from standard monitoring, entropy and SPI data were allowed to be included into the management of anesthesia (ESMG).

### 2.2. Anesthesia

Sedative premedication was prescribed in a dosage of 0.01–0.02 mg/kg midazolam, which was adjusted to the patient’s condition. Before induction of anesthesia, entropy electrodes were applied to the patient’s forehead as recommended by the manufacturer. Anesthesia was induced using propofol or etomidate (depending on the indications) in combination with fentanyl 2–3 µg/kg, followed by a neuromuscular block to facilitate tracheal intubation with rocuronium 0.6–1 mg/kg. Anesthesia was maintained using a volatile anesthetic (sevoflurane). The anesthesiologists were unrestricted in using conventional regimens of opioid analgesics and neuromuscular blocking agents as required. In order to maintain an anesthetic state in the SM Group, anesthesia was adjusted according to somatic response and hemodynamic events, while in the ESM Group, the anesthesiologists tailored anesthesia to achieve state entropy between 40–60 and an SPI value≤ 50.

### 2.3. Data Collection, Assesment of Postoperative of POCD, and Delirium

For each patient included in the ESM Group, we recorded the state entropy (SE) and response entropy (RE) in the awakening state, every 15 min after the beginning of surgery and at extubation time. Because negligible differences exist between state and responsive entropy in curarized patients, we decided to acquire only state entropy data. Other data collected included patient characteristics, surgical procedure, anesthetic data, and the intraoperative hemodynamics.

Between the groups, we recorded and compared the incidence of hypotension, bradycardia, tachycardia (variation of more than 20% from preinduction values of mean arterial blood pressure and heart rate). Patients were discharge from the post-anesthesia care unit based on the modified Aldrete score criteria. Postoperative analgesia was guided according to patient demands and consisted of 1 g paracetamol every 6 h, 20 mg nefopam every 12 h, or morphine (0.1 mg/kg) every 8 h, as well as 50–100 mg ketoprofen every12 h in selected cases.

Postoperative cognitive dysfunctions were assessed 24 h, 48 h, and 72 h after surgery using the NEECHAM Confusion Scale. Patients’ cognitive status could not be further evaluated because the majority of patients were discharged from ICU after 3 days. Another reason for taking into account only the first 3 postoperative days was other postoperative events that could have interfered with our findings. Screening was performed by trained medical personnel.

### 2.4. Statistical Analysis

The objective of this study was to demonstrate that the use of entropy and SPI monitoring in assessing anesthetic depth in emergency surgery is associated with a reduction in postoperative cognitive dysfunctions events. GraphPad 8Prism and MedCalc14.1 were used for statistical analysis.

Given the objective of this study, the correlations between the doses of anesthetics used and the NEECHAM score imposed a sufficient sample size to meet this goal. For this calculation, we used the MedCalc program 14.1 (Sampling-Correlation coefficient). We considered it appropriate to use a significance level of 0.05 to avoid the occurrence of a type 1 error (alpha level 2-sided) and 0.1 to avoid the occurrence of a type 2 error (beta) using an input of the correlation coefficient of 0.5 (the hypothesized or anticipated correlation coefficient). At least 29 patients were required for each group and patients were randomized according to the permuted block technique.

The Anderson–Darling test was used to test the data distribution. Data with normal distribution were compared using the student’s t-test and presented as mean with SD, and data that did not follow the normal distribution were analyzed using nonparametric tests (Mann–Whitney). Different methods for correlation analyses available from MedCalc14.1 were performed, namely Pearson correlation (r) for Gaussian distribution and Spearman rho for nonparametric data. Nominal data were compared using the chi-square test or Fisher’s exact test. A *p*-value < 0.05 was considered statistically significant.

In order identify how we could avoid postoperative cognitive dysfunction, we developed a logistic regression model that used a NEECHAM score at 24 h higher than 24 (indicating the absence of cognitive dysfunction) as a dependent variable. The logistic regression model included the use of entropy monitoring and doses of fentanyl, sevoflurane, and norepinephrine.

## 3. Results

**Figure 1:**
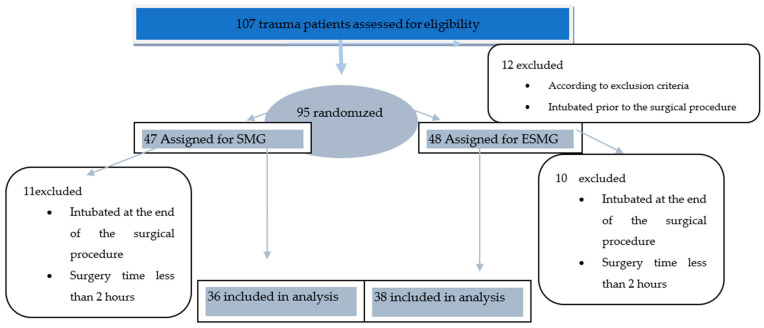
Data collection flowchart.

Of 107 trauma patients undergoing general emergency noncardiac surgery, 12 patients were excluded from the study after application of the exclusion criteria. The remaining 95 patients were assigned using the permuted block randomization design in a 1:1 ratio to the standard monitoring group (SMG) or to the entropy-SPI standard monitoring group (ESMG). Of these patients, 11 and 10 subjects, respectively, were excluded from data analysis either because they remained intubated at the end of the procedure or because the length of the procedure was less than 2 h (Figure 1).

Patient characteristics were similar in both groups (SMG, ESMG; Table 1), and no significant difference was found in the preoperative data except for the duration of anesthesia. Due to the heterogeneity of trauma patients and the variety of surgical procedures employed, no significant statistical analysis could be performed given the small sample for each group studied. Regarding comorbidities, the most frequently associated pathologies were represented by cardiovascular disease (arterial hypertension, ischemic cardiomyopathy) and obesity.

The total dose of fentanyl administered to patients was lower in the ESM Group than in the SM Group, with a statistically significant difference between the two groups(*p* < 0.0001). Sevoflurane uptake per hour was significantly lower in the study group than in the control group (*p* < 0.0001) (Table 2). Anesthesia length was approximately 17 min shorter in the entropy and SPI monitored group than in the standard monitored group (132.52 vs. 150.05 min, *p* = 0.0013). The shorter anesthesia length in the ESM group might be a cofounding factor with regard to the anesthetic volatile consumption.

In regard to intraoperative fluid management, fewer fluids were used for the ESM Group, and statistically significant results between the two groups were found only for crystalloid (*p* = 0.010). As for the noradrenaline dosage, the ESM Group received a smaller dose of vasopressor in comparison to the SM Group (*p* < 0.0001) (Table 2).

Hemodynamic events are listed in Table 3. Intraoperative hypotension was encountered more frequently in the control group (*p* < 0.0001). No statistically significant differences were noted between the two groups regarding the incidence of other hemodynamic disturbances.

### Postoperative Delirium and Cognitive Dysfunction

**Figure 2:**
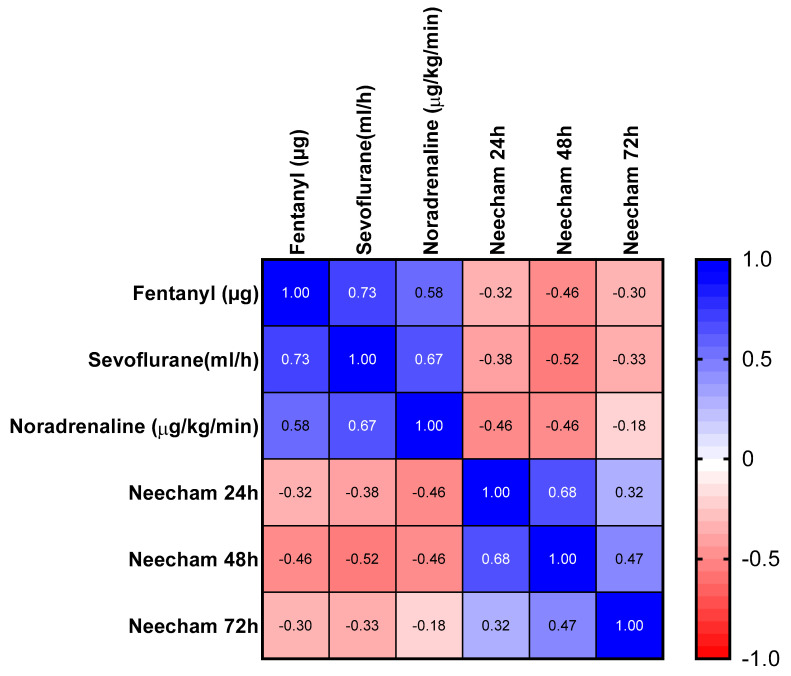
Correlation matrix. The table above shows correlations coefficients between the following variables: Fentanyl (µg), sevoflurane (mL/h), noradrenaline (µg/kg/min) and NEECHAM score at 24 h, 48 h, and 72 h. The colorencodes the sign of correlation between each 2 variables: Blue for positive r values and red for negative r values.

Although fewer patients in the intervention group experienced postoperative cognitive dysfunctions episodes in comparison to the control group, the results were not statistically significant (*p* = 0.08). The study showed a statistically significant inverse correlation between fentanyl and the NEECHAM Confusion Scale at 24 h (r = −0.32, *p* = 0.0005) and 48 h (r = −0.46, *p* = 0.0002), sevoflurane and the NEECHAM Confusion Scale at 24 h (r = −0.38, *p* = 0.0014) and 48 h (r = −0.52, *p* = 0.0002), and noradrenaline and POCD events in the first 48 h (r = −0.46, *p* = 0.0013 for the first 24 h respectively, and r = −0.46, *p* = 0.0002 for the next 24 h) (Figure 2). There was no statistically significant correlation between fentanyl, sevoflurane, or noradrenaline and POCD at 72 h (Figure 2).

In order to identify how we couldavoid postoperative cognitive dysfunction, we developed a logistic regression model that used a NEECHAM score higher than 24 points (indicating absence of cognitive dysfunction) at 24 h as a dependent variable. A four-predictor logistic model was fitted to the data to test the research hypothesis regarding the relationship between the use of entropy and the doses of anesthetic drugs and vasopressor with the advent of postoperative cognitive dysfunctions.

The logistic regression hadan overall model fit described by a nullmodel-2 Log Likelihood of 74.150 and a full model-2 Log Likelihood of 65.311, with a chi-squared value of 8.840 (*p* = 0.06). The goodness of fit of this regression model was calculated with Cox&Snell (R^2^ = 0.19). According to the model, the log of the odds of a patient to develop POCD was negatively related to the dose of fentanyl, sevoflurane, or noradrenaline and positively related toentropy and SPI monitoring (Table 4).

## 4. Discussion

The main finding of this study was that entropy and Surgical Pleth Index-guided anesthesia versus standard monitoring may reduce the incidence of postoperative cognitive dysfunction in the first 72 h for patients undergoing general emergency noncardiac surgery. Also, entropy and SPI may offer a protective role in developing postoperative cognitive dysfunctions. The reported incidence varies greatly in the literature [16,17], especially because neuromonitoring anesthesia has been studied less during emergency noncardiac surgery in comparison to elective surgery.

In our research, we found a substantial reduction in anesthesia duration in the entropy and Surgical PlethIndexmonitored group than in the standard monitored group (132.52 vs. 150.05 min, *p* = 0.0013). The shorter anesthesia length in the ESM Group might be a confounding factor with regard to the anesthetic volatile consumption. In our study, we observed significantly lower sevoflurane doses in the ESM Group. Previous studies demonstrated that neuromonitoring may lead to a less ‘roller-coaster’-like anesthesia [18] and less fluctuation from a defined target than the clinical estimation of anesthetic depth only [19]. Hor et al. conducted a randomized controlled trial in order to assess sevoflurane uptake in patients undergoing major surgery andfounda significant reduction in sevoflurane uptake with the use of entropy, in addition to a faster extubation [20]. Fedorow et al. highlighted that using neuromonitoring in order totitrateanesthetic agents may avoid an unnecessary increase in anesthesialevels and possible neurotoxic effects, especially in high-risk patients [21]. Our data suggest that anesthetic agents may represent a risk factor for developing POCD in the first 48 h. This finding is consistent with previouslypublished studies. According to Micha et al., sevoflurane has a negative influence on short-termcognition [22].

Another pharmacological factor, fentanyl, can be considered a causal factor for the presence of POCD, and we have identified significant dose reduction in the entropy-SPI monitored group in comparison to the standard monitored group. In our study, we identified that fentanyl may represent a risk factor for developing POCD in the first 48 h, but the results cannot be extended topatients who develop POCD in the next 24 h. Although the incidence of cognitive disorders is highly dependent on the type of surgery and general anesthesia management [23,24], opioid treatment remains very influential in POCD occurrence [25].

Emergency surgery is usually closely related to hemodynamic instability. Thus, another favorable trend for the entropy-SPI studied group is represented by fewer hypotensive eventsin the intervention group and by the significantly decreased demand for vasopressor. Intraoperative hypotension was encountered more frequently in the control group (*p* < 0.0001). It is well known that, in addition to uncontrolled anesthetic exposure, another important factor that may increase the risk of developing POCD is represented by blood pressure fluctuation [26,27]. As Wu et al. investigated in a randomized controlled trial, this may be related to sevoflurane consumption [28]. However, we must keep in mind that, in trauma settings, other important factors may contribute to hemodynamic alteration (type of injury, intravascular volemic status, volemic resuscitation, response to tissue injury, tissue perfusion, etc.) [29]. Our findings highlight that noradrenaline may contribute to cognitive impairment in the first 48 h after surgery. Although vasopressors are a cornerstone for treating refractory hypovolemic shock, they may also exhibit negative side effects with harmful repercussion on cerebral perfusion [30,31].

In our study, the majority of patients experiencedthe following comorbidities: Cardiovascular disease (arterial hypertension, ischemic cardiomyopathy) and obesity. Current data do not support the hypothesis that these comorbidities are potential cofounders for developing postoperative cognitive dysfunctions [32,33].

## 5. Limits

Although in the intervention group, fewer patients experienced postoperative cognitive dysfunctions episodes in comparison to the control group, the results were not statistically significant (*p* < 0.08). We consider that one of the main drawbacks of the study was the inability to control all ofthe risk factors that contribute to the development cognitive disorders.

Another study limitation is the consequence of not being to further evaluate postoperative cognitive disorders after 72 h because the majority of patients were discharged from ICU. We also considered that, after 72 h, other factors may interfere with cognitive function and mislead POCD screening.

Due to the fact that neuromonitoring anesthesia and nociception have been studied less frequentlyduring emergency noncardiac surgery in comparison to elective surgery, the available papers do not allow us to entirely compare the magnitude of our findings with the previous published data.

The present research included a small number of patients in each group. In order to establish future relevant knowledge for improving patients’ cognitive outcome, we con-sider it imperative to recruit a higher number of patients.

## 6. Conclusions

The present study was designed to reflect routine clinical practice in emergency settings. It is difficult to isolate one perioperative risk factor for POCD in studies even when excluding individual factors. Despite the extensive research conducted in recent years onthe subject, the causes and pathophysiological mechanismsresponsible for postoperative cognitive decline remain unclear. Entropy and SPI monitoring during anesthesia may play an important role in diminishing the risk ofdeveloping immediate postoperative cognitive dysfunctions after emergency surgery. Also, sevoflurane, fentanyl, and noradrenaline may be closely associated with POCD occurrence in the first 48 h. In order to confirm our hypothesis, we considered that our study required a higher number of patientsto be enrolled. Building upon the data found inour research, we suggest monitoring intraoperative anesthetic depth using entropy and nociception through the SurgicalPlethIndex (SPI) in patients with pre-existing cognitive impairment in order to investigate postoperative cognitive dysfunction in future research.

## Figures and Tables

**Table 1:** Patient characteristics.

		SMG (*n* = 36)	ESMG (*n* = 38)	*p*-Value
Gender—Female/male **		15/21	17/21	
ASA Score *, *n* (%) **	II	12(33.3)	9(23.6)	*p* = 0.582
	III	17(47.2)	18(47.3)	
	IV	7(19.4)	11(28.9)	

* ASA, American Society of Anesthesiologists. ** Categorical data were expressed as number and percentage.

**Table 2:** Comparison of entropy and SPI-guided anesthesia in contrast with standard monitoring-guided anesthesia.

Patient Characteristics	Median Group ESMG	Median Group SMG	95%CI for the Median ESMG	95%CI for the Median SMG	Interquartile Range ESMG	Interquartile Range SMG	*p*-Value
Age	45	44	35.6 to 54.00	36.00 to 57.00	27.5 to 59.5	32.00 to 64.00	0.681
Temperature	36.7	37	36.5 to 36.9	36.7 to 37.1	36.4 to 37.00	36.5 to 37.2	0.024
Fentanyl (µg)	350	500	332.9 to 350.00	450.00 to 500.00	300.00 to 400.00	450.00 to 550.00	<0.001
Sevoflurane(mL/h)	3.2	5.15	3.00 to 3.40	5.00to 5.3	2.9 to 3.6	4.9 to 5.6	<0.001
Crystalloid (mL)	2500	3250	2500.00 to 3170.52	3000.00 to 4000.00	2500.00 to 3500.00	3000.00 to 4000.00	0.010
Colloid (mL)	1000	1000	500.00 to 1000.00	500.00 to 1000.00	500.00 to 1000.00	500.00 to 1000.00	0.324
Noradrenaline (µg/kg/min)	0.08	1	0.05 to 0.50	0.9 to 1.2	0.05 to 0.5	0.8 to 1.3	<0.001

Data were analyzed using the Mann–Whitney test.

**Table 3:** Comparison of entropy and SPI-guided anesthesia in contrast with standard monitoring-guided anesthesia regarding adverse intraoperative hemodynamic events.

	SMG	ESMG	*p*-Value
At least one intraoperatory episode of			
Tachycardia	10	12	0.61
Bradycardia	10	4	0.13
Hypotension	36	18	0.0001

Nominal data were compared using Fisher’s exact test.

**Table 4:** Logistic regression analysis of 74 patients for POCD appearance.

Coefficient and Standard Errors				
**Variable**	**Coefficient**	**Std.Error**	**Wald**	**P**
ESMG = 1	4.1	1.8	5.2	0.022
Fentanyl µg	0.006	0.004	1.7	0.182
Noradrenaline µg/kg/min	0.4	0.7	0.3	0.541
Sevoflurane mL/h	0.7	0.7	1.08	0.296
Constant	−7.4	4.3	2.8	0.090
**Odds Ratios and 95% Confidence Intervals**				
**Variable**	**Odds Ratio**		**95%CI**	
ESMG = 1	66.1		1.8 to 2423.3	
Fentanyl µg	1.006		0.9 to 1.01	
Noradrenaline µg/kg/min	1.5		0.3 to 7.03	
Sevoflurane mL/h	2.1		0.5 to 9.3	

## Data Availability

No data were reported.
